# Configuration of Gut Microbiota Structure and Potential Functionality in Two Teleosts under the Influence of Dietary Insect Meals

**DOI:** 10.3390/microorganisms9040699

**Published:** 2021-03-28

**Authors:** Nikolas Panteli, Maria Mastoraki, Maria Lazarina, Stavros Chatzifotis, Eleni Mente, Konstantinos Ar. Kormas, Efthimia Antonopoulou

**Affiliations:** 1 Department of Zoology, School of Biology, Aristotle University of Thessaloniki, 54124 Thessaloniki, Greece; nkpanteli@bio.auth.gr (N.P.); mmastora@bio.auth.gr (M.M.); 2 Department of Ecology, School of Biology, Aristotle University of Thessaloniki, 54124 Thessaloniki, Greece; mlazarin@bio.auth.gr; 3 Institute of Marine Biology, Biotechnology and Aquaculture, Hellenic Centre for Marine Research, Gournes Pediados, 71003 Heraklion, Greece; stavros@hcmr.gr; 4 Department of Ichthyology and Aquatic Environment, School of Agricultural Sciences, University of Thessaly, 38446 Volos, Greece; emente@uth.gr (E.M.); kkormas@uth.gr (K.A.K.)

**Keywords:** gut microbiota, *Sparus aurata*, *Dicentrarchus labrax*, metagenomics, 16S rRNA, sustainable aquaculture, alternative animal feeds, *Hermetia illucens*, *Tenebrio molitor*, *Musca domestica*

## Abstract

Insect meals are considered promising, eco-friendly, alternative ingredients for aquafeed. Considering the dietary influence on establishment of functioning gut microbiota, the effect of the insect meal diets on the microbial ecology should be addressed. The present study assessed diet- and species-specific shifts in gut resident bacterial communities of juvenile reared *Dicentrarchus labrax* and *Sparus aurata* in response to three experimental diets with insect meals from three insects (*Hermetia illucens*, *Tenebrio molitor*, *Musca domestica*), using high-throughput Illumina sequencing of the V3–V4 region of the 16S rRNA gene. The dominant phyla were Firmicutes, Proteobacteria and Actinobacteria in all dietary treatments. *Anaerococcus* sp., *Cutibacterium* sp. and *Pseudomonas* sp. in *D. labrax*, and *Staphylococcus* sp., *Hafnia* sp. and *Aeromonas* sp. in *S. aurata* were the most enriched shared species, following insect-meal inclusion. Network analysis of the dietary treatments highlighted diet-induced changes in the microbial community assemblies and revealed unique and shared microbe-to-microbe interactions. PICRUSt-predicted Kyoto Encyclopedia of Genes and Genomes (KEGG) pathways were significantly differentiated, including genes associated with metabolic pathways. The present findings strengthen the importance of diet in microbiota configuration and underline that different insects as fish feed ingredients elicit species-specific differential responses of structural and functional dynamics in gut microbial communities.

## 1. Introduction

Edible insects are slowly making their appearance in Western culture [1] as a proposal to tackle the rapid growth of the population, which is expected to reach 9.8 billion by 2050 (UN, 2017). More than 1900 insect species throughout the world have been an integral part of the human diet for centuries [2]. Among the many advantages that accompany the utilization of insects as an alternative source of edible protein is the high content and high nutritional value of protein [2,3], the adequate profile of amino acids [3], and the high feed conversion ratio [4]. In addition, most insect species are characterized by high fecundity and multivoltinism [4], ensuring a rapid yet more environmentally sustainable mass production, which requires similar amounts of energy but emits much less greenhouse gas compared to conventional livestock production [5,6]. Consequently, insects may prove to be the immediate solution to global food security both as food for human consumption and as feed for livestock and fish.

The aquaculture industry is undergoing vigorous efforts to decrease the use of fish meal as the basal protein source for aquafeed due to economic constraints and environmental exploitation of forage fish [7]. Hence, partial or total dietary supplementation of fish meal with eco-friendly feed ingredients has been under constant review in recent years. Plant-based meals, particularly soybean [8,9,10,11], have been studied extensively as a substitution for fish meal given the competitive price and reasonably balanced amino acid profile [12]. However, reduction in growth performance [8] and feed intake [13], in addition to negative-associated gut microbial imbalances [9,14,15] have been described. As the search for ideal aquafeeds based on alternative protein sources is strenuously ongoing, insects have recently emerged as potential ingredients [16,17].

A challenging problem that arises in response to fish meal replacement is diet-elicited effects on various biological processes. Plenty of studies have reported modifications of growth performance [18,19], metabolism [20,21], and humoral and cellular immunology [19,22,23] due to changes in the dietary composition. Advancements in Next-Generation Sequencing have enlightened the cognition of microbial communities inhabiting a variety of environments, including fish gut microbiota. However, few studies have focused to date on the imminent impact of insect meal on gut microbial communities [17,24,25,26]. Health promotion is profoundly linked to the state of gut microbiota [27] given its pronounced influence on gut nutrient absorption and metabolism [28,29], gut epithelium differentiation [30], immunity, and prevention of pathogens’ colonization [31,32,33]. Dysbiosis of gut microbiota may facilitate microbial translocation, consequently inducing inflammation [34].

Considering the above challenges, any approach of utilizing insects in aquafeeds should therefore ensure the maintenance of balanced microbiota that contributes to fish welfare and production optimization. With the exception of Antonopoulou et al. [17], the effects of insects as aquafeed ingredients on the microbial communities of European sea bass *(Dicentrarchus labrax)* and gilthead sea bream (*Sparus aurata*) have not been studied in depth despite their significance in the Mediterranean aquaculture. Using these two fish species as hosts and the same partial fish meal substitutes in aquafeeds, i.e., mealworm (*Tenebrio molitor*), black soldier fly (*Hermetia illucens*), and housefly (*Musca domestica*) larvae, this study investigated their gut microbial profiling and functionality. This study represents the first comprehensive report on diet-specific and species-specific shifts of gut microbial communities in response to identical dietary inclusion of insects as fish meal substitutes.

## 2. Materials and Methods

### 2.1. Ethical Approval

The experiments were authorized by the ethics committee of the region of Crete, General Directorate of Agricultural and Veterinary, under the updated license No 255340/01.03.2017. The experimental procedures were conducted in accordance with the guidelines of legislation of the Greek Presidential Degree No 56/2013 and Official Journal of the Greek Government No. 106/30 April 2013 on the protection of animals used for scientific purposes. Scientists involved in animal handling and sampling were accredited by the Federation of Laboratory Animal Science Associations (FELASA) in categories A–D of competence.

### 2.2. Experimental Conditions and Feed Formulation

The experiments were carried out at the Institute of Marine Biology, Biotechnology and Aquaculture (IMBBC) of the Hellenic Centre for Marine Research (Heraklion, Crete, Greece). Juvenile *D. labrax* and *S. aurata* of an average weight of 5.7 ± 1 g and 29.5 ± 0.7 g, respectively, obtained from the IMBBC hatchery, were randomly allocated into twelve indoor 250 L tanks. A detailed description of the experimental procedure of *D. labrax* is given in Mastoraki et al. [35]. Prior to the experimental trial, juveniles were acclimatized for a short period of time to the experimental conditions. The tanks were consistently supplied with borehole water through an open circulation system with a water renewal of 200% per hour and oxygen saturation over 80%. Throughout the experiment, the water temperature was adjusted to 19.3 ± 0.2 °C for *D. labrax* and 19.9 ± 0.1 °C for *S. aurata*, and juveniles were exposed to a natural 12:12 light regime.

Prior to diets’ formulation, all insects purchased from different sources underwent drying and/or homogenization processes. Four isoproteic and isoenergetic diets were designed in compliance with the nutritional specifications for the two teleost species using commercial ingredients (Supplementary Material Table S1). The control diet (FM) contained 65% fish meal as the sole protein source, whereas the three experimental diets were formulated with 30% substitution of fish meal with insect meals from larvae of *T. molitor* (TM), *H. illucens* (HI), or *M. domestica* (MD). The proximate analysis of the experimental diets was calculated using the composition of the ingredients. During an experimental period of three months, fish were hand-fed to satiation, three times a day, seven days a week. Upon termination of the feeding trial, the fish were fasted for 24 h. Growth performance parameters and somatometric indices were calculated as described in the Supplementary Material.

### 2.3. Midgut Sample Collection

As described extensively in our previous study [36], sufficient biological replication is required in order to yield statistically secure inferences in dietary metabarcoding studies. Thus, a sample size of twelve fish per diet was considered capable of capturing the diet-induced shifts in microbial communities for each dietary treatment [36]. For each teleost species, twelve healthy fish per dietary treatment (four fish per tank) were randomly sampled and euthanized by anesthesia overdose using phenoxyethanol. The gastrointestinal tract was dissected, and the midgut was aseptically removed with sterile forceps. Considering that our study aimed for the autochthonous microbial populations, the removal of indigestible food residues and subsequently exclusion of non-adherent microbes was achieved by gently applying mechanical force, and by cleansing three times in sterile particle-free (<0.2 μm) distilled water (SPFDW) as described in Kormas et al. [37]. Dissecting tools were alcohol sterilized anew between different samples. Midgut tissues were collected in sterile tubes and kept on ice prior to storage at −80 °C until DNA extraction.

### 2.4. DNA Extraction and 16S rRNA Gene Sequencing

DNA was extracted from individual midgut samples of 25 mg using the QIAamp DNA Mini Kit (QIAGEN, Hilden, Germany) according to the manufacturer’s protocol. To avoid contamination, equipment was sterilized among samples throughout the process. The yield and integrity of the extracted DNA samples were determined using NanoDrop TM-1000 spectrophotometer (Thermo Scientific, Wilmington, DE, USA) prior to storage at −80 °C until further analysis.

The V3-V4 region of the 16S rDNA gene was amplified and sequenced on the MiSeq Illumina platform using the primer pair S-D-Bact-0341-b-S-17 (5′-CCTACGGGNGGCWGCAG-3′) and S-D-Bact-0785-a-A-21 (5′-GACTACHVGGGTATCTAATCC-3′) [38]. Library preparation and sequencing were implemented at the MRDNA (Molecular Research) Ltd. (Shallowater, TX, USA) sequencing facilities. Polymerase Chain Reaction (PCR) of 30 cycles was performed with HotStarTaq Plus Master Mix Kit (Qiagen, Valencia, CA, USA) under the following conditions: denaturation at 94 °C (3 min); 28 cycles of 94 °C (30 s), 53 °C (40 s), and 72 °C (1 min); and a final extension at 72 °C (5 min).

### 2.5. Sequencing Data Processing

Raw reads obtained from sequencing were processed and analyzed using the MOTHUR software (v. 1.36.0) [39] according to the steps described in the MiSeq Standard Operating Procedure (SOP) pipeline. Briefly, after trimming barcodes and primer sequences, quality control was performed through the ‘screen.seqs’ command and sequences were removed according to the following filtering parameters: length less than 250 bp, ambiguous bases, average quality score less than 25, and homopolymers longer than eight nucleotides. Thereafter, the remaining sequences were aligned against the SILVA 119 database [40] and the screen.seqs command was re-run in order to ensure that sequences overlap the specific region. Chimeric reads were filtered from subsequent analyses using the VSEARCH algorithm. Sequences were clustered into operational taxonomic units (OTUs) based on the average neighbor algorithm at a 97% sequence identity threshold [41,42]. High-quality OTU sequences were classified to different taxonomic levels using the SILVA 119 database [40] with confidence value set above 80%. The average number of quality-controlled classifiable reads for *D. labrax* was 87,089 ± 9405, 71,679 ± 8003, 54,279 ± 4528, and 53,016 ± 3457 in FM, HI, TM, and MD treatment, respectively. Regarding *S. aurata*, the average number of reads was 27,900 ± 3499 for FM, 21,269 ± 3702 for HI, 29,348 ± 3014 for TM, and 20,260 ± 1543 for MD. Due to differences in sequencing depth between *D. labrax* and *S. aurata*, the samples were normalized using the ‘normalize.shared’ command.

### 2.6. Statistical and Bioinformatics Analysis

To estimate and compare diversity of gut microbial communities, Shannon diversity index, Simpson reciprocal index of diversity (1-D), Chao1 richness estimator, and rarefaction curves were calculated. Statistical and diversity analyses were carried out using the PAST 3.25 software [43]. Nonmetric Multidimensional Scaling (NMDS) analyses based on the Bray–Curtis dissimilarity index and Jaccard index were conducted in the R environment with the vegan package. The structure-based Bray–Curtis distance weights on the relative abundances of the OTUs, whereas Jaccard distance estimates dissimilarities based on the presence or absence of OTUs. Prediction of microbes’ functional profiling was carried out through the software package PICRUSt (Phylogenetic Investigation of Communities by Reconstruction of Unobserved States) [44] with the type of functional predictions set to KEGG Orthologs (Kyoto Encyclopedia of Genes and Genomes). PICRUSt results were explored with Statistical Analysis of Metagenomic Profiles (STAMP) [45] software in order to identify significant differentiation in microbial function abundances in response to fish meal substitution. Moreover, to avoid multicollinearity, Variance Inflation Factor analysis was conducted using the usdm package in the R environment and variables with a score >10 were excluded. Spearman’s rho non-parametric correlation was performed to explore the relationships between gut bacterial content (e.g., richness, bacterial phylum ratios) and somatometric indices and growth performance parameters. Correlations were based on individual data per diet. The statistical significance threshold was predetermined at the *p <* 0.05 level.

### 2.7. Co-occurrence Network Analysis

Furthermore, a co-occurrence network analysis was conducted in the R environment with the vegan and igraph packages following a previously published script [46]. To compare the co-occurrence patterns of bacterial genera, four networks were generated based on the four dietary treatments, including genera derived from the two teleost species, in addition to an overall network for both species based on all diets. All pairwise Spearman’s correlations between bacterial OTU abundance in gut samples were calculated. The correlations were considered to be statistically robust when the Spearman’s correlation coefficient (r) was >0.7 (r < −0.7) and the FDR-adjusted *p*-value was ≤0.001. Based on the aforementioned correlations, networks were constructed with nodes indicating bacterial genera and edges representing the significant correlation (positive or negative interactions) among the nodes. Furthermore, a set of descriptive measures (number of nodes and edges, average path length, network diameter, average degree, graph density, clustering coefficient, and modularity) was calculated for each network. Based on betweenness centrality (BC), which is a measure of the centrality of a node in a network, genera that can be considered as network keystone species were identified. As described in Gonzalez et al. [47], a high BC score is an indicator of keystone species, integral for maintaining connectivity within the network.

## 3. Results

### 3.1. Growth Performance and Somatic Indeces

After the feeding trial, partial fish meal substitution with insect meals (HI, TM, MD) did not induce alterations in growth performance and nutrient utilization of *D. labrax* and *S. aurata* used in the present study. As shown in Table S2 in Supplementary Material, no significant changes in final body weight (FBW), weight gain (WG), daily feed intake (DFI), specific growth rate (SGR), and feed conservation ratio (FCR) were observed among the dietary treatments. During the experiment, no mortality was recorded.

### 3.2. Effects of Insect Meals on Gut Microbiota Diversity and Composition

**Figure 1:**
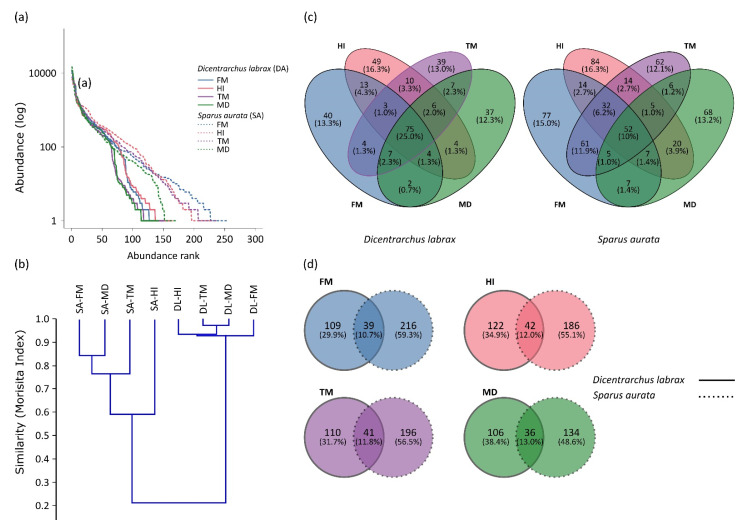
Gut microbiota of *Dicentrarchus labrax* and *Sparus aurata* fed control (FM: fish meal) and insect-containing diets (HI: *Hermetia illucens*; TM: *Tenebrio molitor*; MD: *Musca domestica*). (**a**) Comparison of gut microbial community structure among the four dietary treatments in the two teleost species. Rank-abundance curves were constructed based on abundance derived from samples (*n* = 12) for each diet. Curves for different diets and species are represented by different colors and lines, respectively. (**b**) Cluster analysis based on the Morisita similarity index of the bacterial operational taxonomic units (OTUs) derived from the gut of *D. labrax* and *S. aurata* fed control and insect-containing diets. Bootstrap analysis with 1000 replicates was conducted. (**c**) Venn diagrams displaying shared and unique bacterial OTUs between the four diets in *D. labrax* and *S. aurata*. (**d**) Venn diagrams displaying shared and unique bacterial OTUs between *D. labrax* and *S. aurata* fed the same diet. The percentages in the Venn diagrams indicate the respective ratios to the total number of bacterial OTUs.

Following standardization of different samples in order to allow between-species comparisons, 733 unique OTUs at 97% identity were obtained. Rarefaction curves approached a plateau in most samples indicating sufficient sequencing depth (Supplementary Material Figure S1). *D. labrax* and *S. aurata* displayed the highest species richness in the FM diet (58.4 ± 2.2 and 54.2 ± 7, respectively), whereas MD showcased the lowest number of bacterial OTUs (55.1 ± 3.2 and 37.8 ± 3.3, respectively). Compared to the other diets, MD displayed a decrease regarding OTU richness in *S. aurata*. No significant differences were observed among tanks in each dietary treatment. Rank abundance curves (Figure 1a) indicated that *S. aurata* ranges were wider than those of *D. labrax*, whereas the pattern of few abundant OTUs and numerous OTUs at low abundance was apparent in all dietary treatments of both species. The number of OTUs resulting in a cumulative relative dominance of over 80% of total relative abundance is presented in Table S3 in Supplementary Material.

**Figure 2:**
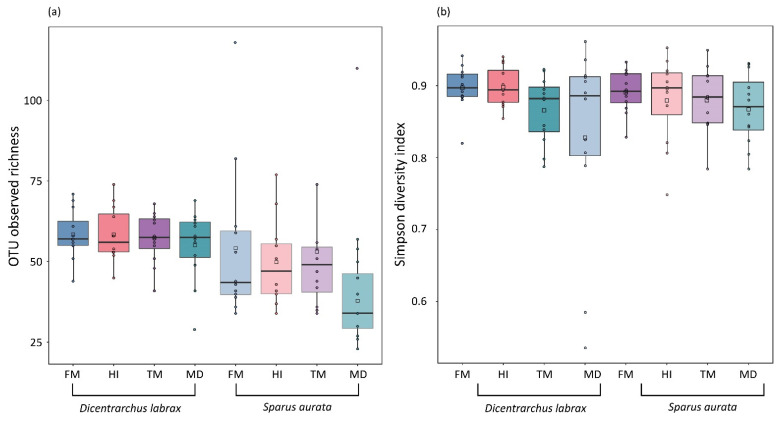
Scattered boxplots of (**a**) observed bacterial OTU richness and (**b**) Simpson reciprocal index (1-D) of gut microbiota among *Dicentrarchus labrax* and *Sparus aurata* fed control (FM) and insect-containing diets (HI, TM, MD). Abbreviations according to Figure 1.

**Figure 3:**
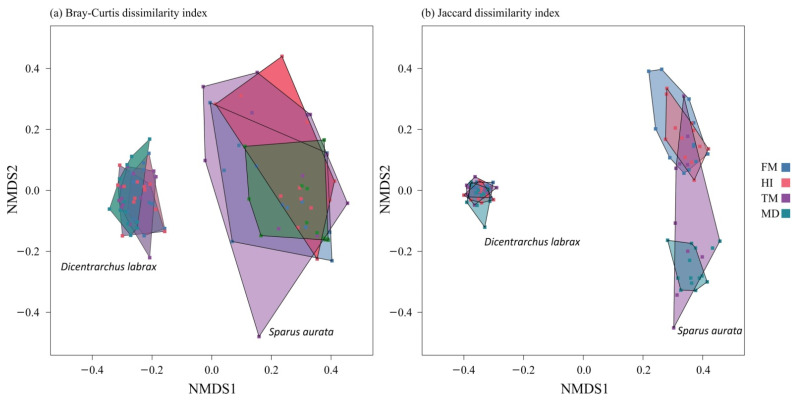
Nonmetric multidimensional scaling (NMDS) plots of the gut bacterial communities of *Dicentrarchus labrax* and *Sparus aurata* fed control (FM) and insect meal-containing diets (HI, TM, MD), derived from (**a**) Bray–Curtis dissimilarity index and (**b**) Jaccard dissimilarity index. Different colors indicate different diets. Abbreviations according to Figure 1.

The structure of gut microbial communities was observed through diversity analysis. OTU richness and the Simpson diversity index revealed no significant differences in *D. labrax* or *S. aurata*, regardless the diet (Figure 2). Concerning *S. aurata*, the Chao1 estimator presented significant variation between the four diets (Kruskal–Wallis test: *p <* 0.01), with MD treatment displaying a significant lower value compared to the other treatments (Mann–Whitney test: *p <* 0.01) (Supplementary Material Table S3). Cluster analysis was performed based on the Morisita similarity index (Figure 1b). Species composition in *D. labrax* microbial communities is highly similar (>90%) in all dietary treatments. However, within the *S. aurata* cluster, HI treatment deviated from the rest, presenting at the lowest similarity value (60%). Differences in the bacterial communities among the two teleosts were also apparent in the ordination plots of NMDS analysis (Figure 3). In comparison to *S. aurata*, *D. labrax* displayed higher over-lapping and similarities between the four dietary treatments. In contrast, based on the Jaccard index community structure in *S. aurata*, the FM and HI group diverged from TM and MD due to the presence/absence of specific OTUs. Moreover, a higher inter-individual variability was observed in TM group of *S. aurata*.

Venn diagrams were constructed for counting the number of unique and common OTUs among dietary treatments and among the two teleost species sharing the same diet (Figure 1c,d, respectively). The percentage of OTUs shared among the four dietary groups was 25% and 10.1% for *D. labrax* and *S. aurata,* respectively. Both examined species displayed an increase in the number of unique OTUs (non-present in any of the other diets) in the HI group compared to FM, whereas a reduction in TM and MD groups was observed. Common OTUs between *D. labrax* and *S. aurata* reached 10.7% in FM, 12% in HI, 11.8% in TM, and 13% in MD groups.

**Figure 4:**
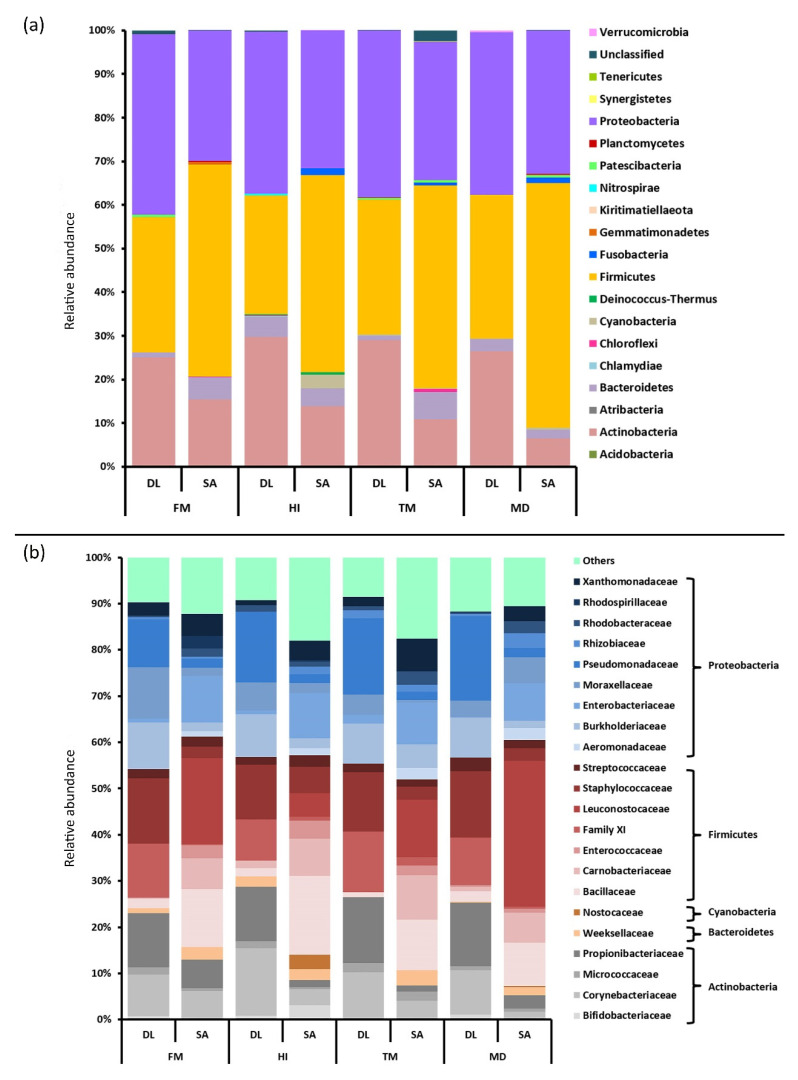
Taxonomic composition of the gut microbial community across the four dietary treatments in *Dicentrarchus labrax* and *Sparus aurata*. Stacked bar chart representing the relative abundance of the different bacterial (**a**) phyla and (**b**) families in the gut of *D. labrax* and *S. aurata* fed control (FM) and insect diets (HI, TM, MD). Each bar displays the microbial community of 12 individuals fed a specific diet. Bacterial operational taxonomic units (OTU) that could not be classified at phylum level were grouped as ‘unclassified’. Bar charts demonstrate bacterial families with an overall abundance of >1%. Color legends represent different phylogenetic groups. Abbreviations according to Figure 1.

The dominant phyla in *D. labrax* were Proteobacteria, Firmicutes, and Actinobacteria across the four diets (Figure 4a). Regardless of the diet, Firmicutes was the most abundant bacterial phylum in the gut microbiota of *S. aurata*, followed by Proteobacteria and Actinobacteria, although the latter displayed lower relative abundance in comparison to *D. labrax*. Compared to the FM diet, the two teleost species displayed the highest elevation of Firmicutes’ relative abundance in MD treatment and the lowest in HI. Partial fish meal substitution affected inversely the relative abundance of Actinobacteria in the two teleost species as an increase was observed in insect-fed *D. labrax* and a decrease in *S. aurata* of HI, TM, and MD groups. Moreover, Bacteroidetes relative abundance was differently altered in *D. labrax* and *S. aurata* in response to the insect meal diets. It is noteworthy that Cyanobacteria was the fifth most abundant phylum in HI dietary treatment displaying a dramatic increase compared to the control diet. Despite the fact that certain OTUs may have initially been identified as members of Cyanobacteria, it must be noted that a novel candidate phylum, i.e., Melainabacteria which is believed to share a common non-photosynthetic, anaerobic, and obligately fermentative ancestor with Cyanobacteria [48], has been proposed as a component of the gut bacterial community. Shifts in the relative abundance led to differentiation of major phyla ratios. Proteobacteria-to-Bacteroidetes and Firmicutes-to-Bacteroidetes ratios were observed among the most pronounced changes in *D. labrax*, which showcased an up to a five-fold decrease in HI and MD dietary groups. In contrast, the abovementioned ratios demonstrated an increase in HI and MD *S. aurata*, and a reduction was observed in TM.

The taxonomic profile at the family level displayed species-specific differences among the two teleosts (Figure 4b). In particular, the most dominant families were Staphylococcaceae and Pseudomonadaceae in *D. labrax* and Bacillaceae and Leuconostocaceae in *S. aurata*. However, within-species differences in abundance were apparent in some families, e.g., Carnobacteriaceae in HI and MD group of *D. labrax* and Rhizobiaceae in all insect meal diets of *S. aurata*. At a finer taxonomic classification, *Staphylococcus* and *Cutibacterium* were the most abundant genera along the *D. labrax* lumen in the four dietary groups, with *Cutibacterium* outnumbering *Staphylococcus* in the TM group. In relation to *S. aurata*, rearrangements were observed regarding the dominant genera among the diets. *Weissella* was predominant in FM and MD dietary treatments, followed by *Bacillus* and *Leuconostoc,* respectively. However, dietary inclusion of HI and TM led to *Enterobacter* and *Bacillus,* respectively, superseding *Weissella* as the most abundant genera, followed by *Atopostipes*.

To further estimate changes in gut microbial communities, differences in bacterial genera between dietary treatments were calculated using the non-parametric factorial Kruskal–Wallis test. In *D. labrax*, significant changes (*p <* 0.05) occurred in five genera (*Acinetobacter*, *Cloacibacterium*, *Diaphorobacter*, *Sphingomonas*, and *Stenotrophomonas*). Regarding *S. aurata*, 26 genera (*Acidiphilium*, *Acidovorax*, *Acinetobacter*, *Aeriscardovia*, *Bacteroides*, *Blastococcus*, *Carnobacterium*, *Cetobacterium*, *Cloacibacterium*, *Curtobacterium*, *Escherichia-Shigella*, *Lawsonella*, *Leuconostoc*, *Methylobacterium*, *Microbacterium*, *Moheibacter*, *Oceanobacillus*, *Ornithinibacillus*, *Paracoccus*, *Ruegeria*, *Skermanella*, *Sphingomonas*, *Streptomyces*, *Terrisporobacter*, *Tyzzerella 4*, and *Weissella*) were differentiated significantly (*p <* 0.05) between the four diets.

In addition, shared bacterial OTUs across all dietary treatments and all samples were determined for each teleost species. Although all insect diets were able to enhance several shared bacterial species, MD exhibited the highest retention or increase. Among the shared bacterial OTUs, OTU004 (closest relative *Anaerococcus* sp.), OTU002 (c.r. *Cutibacterium* sp.) and OTU007 (c.r. *Pseudomonas* sp.) were the most enriched in *D. labrax* following dietary substitutions with HI, TM, and MD, respectively. Enrichment of taxa belonging to the bacterial genus *Staphylococcus* (OTU001), *Hafnia* (OTU654), and *Aeromonas* (OTU655) was observed in *S. aurata* belonging to the dietary groups of HI, TM, and MD, respectively.

### 3.3. Effects of Insect Meals on Functional Profiling of Gut Microbial Communities

**Figure 5:**
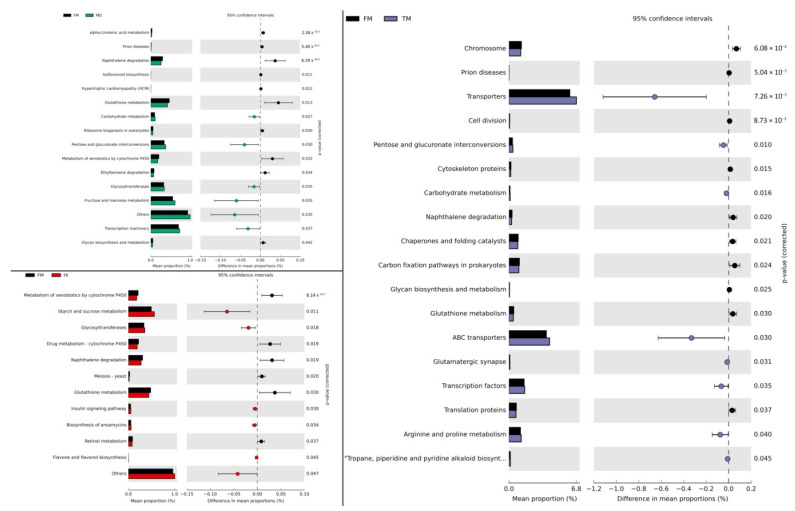
Significantly altered bacterial Kyoto Encyclopedia of Genes and Genomes (KEGG) pathways at level 3 (Welch’s *t*-test, *p* < 0.05) between fish meal (FM) and insect meal (HI, TM, MD) diets fed to *Dicentrarchus labrax*. Predicted functional pathways based on differential abundance were generated by PICRUSt and STAMP analyses. Significant differences were calculated using the Welch’s inverted confidence interval method. Abbreviations according to Figure 1.

**Figure 6:**
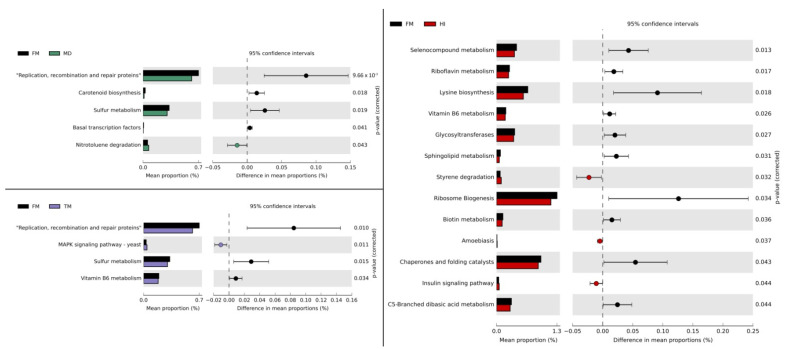
Significantly altered bacterial Kyoto Encyclopedia of Genes and Genomes (KEGG) pathways at level 3 (Welch’s *t*-test, *p* < 0.05) between fish meal (FM) and insect meal (HI, TM, MD) diets fed to *Sparus aurata*. Predicted functional pathways based on differential abundance were generated by PICRUSt and STAMP analyses. Significant differences were calculated using the Welch’s inverted confidence interval method. Abbreviations according to Figure 1.

Furthermore, to determine any functional enrichment or diminishment of gut microbiota over fish meal substitution, functional assessment through PICRUSt was performed to analyze the KEGG pathway compositions in microbial populations. Genes related to membrane transport were the predominant gene family in all diet groups of both species. Several bacterial functional pathways were differentiated significantly (Kruskal–Wallis test: *p <* 0.05) in response to the insect meal administration, as demonstrated in Figure 5 and Figure 6 for *D. labrax* and *S. aurata*, respectively. Concerning *D. labrax*, several functional categories related to metabolic pathways were up-regulated, as such glycan biosynthesis and metabolism (e.g., glycosyltransferases) in HI and MD dietary groups, carbohydrate metabolism in HI, TM, and MD, and amino acid metabolism in TM. However, other metabolism-related genes displayed a significant reduction in comparison to FM, such as genes associated with xenobiotics’ biodegradation and metabolism. A similar down-regulation of functional categories related to metabolic pathways was observed in *S. aurata,* including energy metabolism (e.g., sulfur) in TM and MD dietary treatments, metabolism of cofactors and vitamins (e.g., vitamin B6, riboflavin) in TM and HI, and lipid metabolism in HI. In contrast to *D. labrax*, genes associated with xenobiotics’ biodegradation and metabolism were up-regulated in *S. aurata* following dietary inclusion of MD and HI.

### 3.4. Correlation between Gut Microbiota and Growth Performance

Considering the limited number of studies, e.g., [49], a correlation between indices of fish growth and performance with gut microbiota was performed. The relationships between richness and ratios among major phyla and growth performance parameters are presented in Table S4 for *D. labrax* and Table S5 for *S. aurata* in Supplementary material. The ratios of Firmicutes-to-Bacteroidetes and Proteobacteria-to-Bacteroides were positively correlated with feed conversion ratio, and negatively correlated with weight gain in the control group of *D. labrax*. However, the aforementioned correlations were not maintained in *D. labrax* following the dietary inclusion of insect meals.

### 3.5. Diet-Specific Co-occurrence Networks

**Figure 7:**
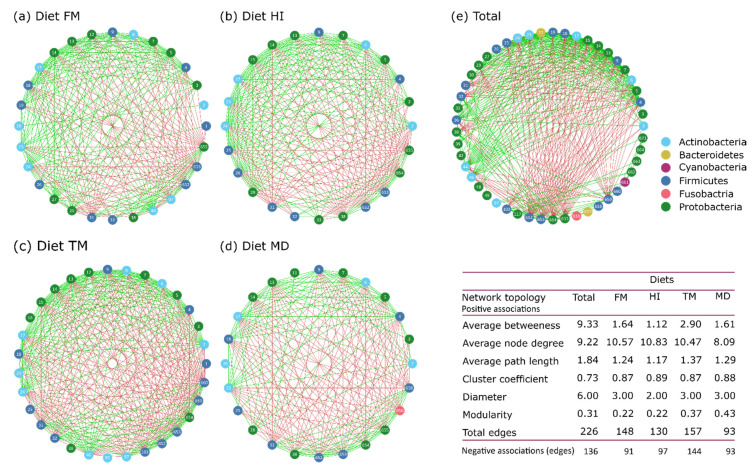
Empirical microbial networks describing the co-occurrence patterns and interactions between bacterial genera in the regime of the four dietary treatments (**a**) FM, (**b**) HI, (**c**) TM, and (**d**) MD based on OTUs derived from the gut of both *Dicentrarchus labrax* and *Sparus aurata*, in addition to a general network (**e**) based on all samples. Each node in the network signifies one bacterial OTU and each edge indicates significant interactions among the nodes, based on Spearman’s correlations (r > 0.7, *p <* 0.001). The green colored edges representpositive interactions and the red colored edges representnegative interactions. Colors of the nodes represent the phylum assigned to each node and are described in the legend. The major topological features of the respective networks are presented in the table. Abbreviations according to Figure 1.

To elucidate whether the diets can favor the interactions among specific OTUs and can contribute to stable community structures within gut microbiota, co-occurrence patterns among each dietary treatment were analyzed using network analysis. Diet-specific microbial networks for both teleosts and their respective major topological properties are presented in Figure 7. Differences in the network size were apparent, because TM displayed the largest network (31 nodes and 301 edges), whereas the smallest network (24 nodes and 186 edges) occurred in MD. Based on the average node degree and total edges, the MD network displayed the lowest microbial network complexity among diet microbial networks, whereas HI was the most connected with an average node degree of 10.83. Modularity was identical between control and HI networks (0.22) and higher for TM and MD networks, whereas the clustering coefficient was preserved at a high level among the four dietary networks, indicating that the neighbors of each node were well connected. Furthermore, the positive correlations outnumbered the negative correlations in all of the networks, with the exception of the MD network, which displayed an equal number of positive and negative correlations. Based on the pairwise Spearman’s correlations, 28 and 16 significant positive and negative correlations, respectively, were shared among the four dietary treatments, whereas insect meals displayed 12 and 13 unique positive and negative correlations, respectively, that were not observed in the control diet. In addition, the nodes were assigned to bacterial taxa, with three major phyla (Actinobacteria, Firmicutes, and Proteobacteria) attributing to most of the networks. Proteobacteria and Firmicutes contributed with the highest number of nodes across all of the networks, with Firmicutes surpassing Proteobacteria in the TM network. In terms of betweenness centrality, which highlights the dynamic of a node on other nodes interactions in the network, the top genera identified as keystone nodes were *Glutamicibacter* (BC = 19.14) for FM, *Acinetobacter*, *Janibacter*, and *Peptoniphilus* (BC = 3.5) for HI, *Delftia* (BC = 18.00) for TM, and *Acinetobacter* (BC = 7.38) for MD.

## 4. Discussion

As nutrition is one of the pivotal key factors that determine the configuration of the gut microbial communities, the main objective of the study was to assess the influence of insect meal administration in two teleosts’ gut microbiota and the diet-specific and/or species-specific shifts in microbial communities. The results reported herein are the first to elucidate the apparent differentiations in structure, composition, and potential functionality of *D. labrax* and *S. aurata* gut microbiota in response to fish meal substitution with insect meal from *T. molitor*, *H. illucens*, and *M. domestica* larvae. Foremost, the two teleost species accepted the insect meal diets with no negative effects on survival, growth performance, and digestibility. Our results revealed that the incorporation of these insects into fish feeds can enrich and enhance the presence of beneficial bacterial species and may rearrange the bacterial hierarchy leading to changes in the concomitant gut microbial ecology.

### 4.1. Gut Microbiota Structure and Composition

Overall, in the present study, differentiation among the gut microbial communities of the two teleosts was apparent. Even sympatrically farmed fishes, including *D. labrax* and *S. aurata*, have been reported to have a species-specific gut microbiota, regardless of similar environmental conditions [50]. Herein, *D. labrax* gut microbial communities in the four dietary groups showcased more similarities, whereas a divergence among dietary groups in *S. aurata* was observed. Bacterial diversity and richness were not influenced by the fish meal substitution, with the exception of *M. domestica* in *S. aurata*. In a similar study [17], despite no significant changes in the diversity indices following partial substitution of fish meal with *T. molitor* meal, the number of bacterial OTUs was increased in the gut of *D. labrax* and *S. aurata*. However, such differences may be attributed to the higher percentage (50%) of *T. molitor* inclusion in the diets, compared to the 30% substitution in this study.

Fish gut microbial profiles tend to differ within species [51,52] and between species [53,54] due to the great influence exerted by miscellaneous factors including diet [55], life stage [56], and water [57], in addition to host selection and phylogeny [58,59]. Sampling of the water microbiota was deemed unnecessary in this study as it would not be representative of the range of the influence exerted on the two species. This is due to the fact that the life cycle of *D. labrax* and *S. aurata* extends over many months during which the marine bacterioplankton is subjected to temporal variances [60,61,62]. In addition, many studies have highlighted the absence of a significant relationship between gut and water microbiota [15,56,63,64,65].

Herein, the dominance of Actinobacteria, Firmicutes, and Proteobacteria as the main gut bacterial residents in both teleosts across all four experimental diets is consistent with previously published studies on microbial communities of fishes [26,55,66], including *D. labrax* and *S. aurata* [15,17,67]. The latter reinforces the possible existence of core microbiota among a plethora of fish species, as previously reported [68], which was able to be maintained despite the dietary inclusion of insect meal. Simultaneously, the dominance of Proteobacteria in *D. labrax* of the control group and the subsequent enhancement of Actinobacteria abundance following the dietary inclusion of *H. illucens* are compatible with those of Huyben et al. [25] and Terova et al. [26] derived from similar studies in *Oncorhynchus mykiss*. However, no such observations were recorded in *S. aurata,* and, additionally, both species displayed a decrease in Firmicutes’ abundance in response to the fish meal substitution with *H. illucens,* which is contrary to the results in *O. mykiss*. Despite this, the dominance of Firmicutes noted in all dietary groups of *S. aurata* is in line with previous studies [69]. An increased proportion of Staphylococcaceae, which was observed in both teleosts fed with the *H. illucens* diet, is in agreement with that of Terova et al. [26] in the gut of *O. mykiss*.

Such dietary-induced shifts in gut microbial ecology may disturb interactions among major phyla [14,17,55,70], thus leading to potential implications in fish physiology. The Firmicutes-to-Bacteroidetes ratio is often regarded as an indicator of healthy human gut microbiota [71]. Regarding the teleosts, it has been indicated that a higher weight gain in grass carp (*Ctenopharyngodon idella*) may be related to a reduced Bacteroidetes-to-Firmicutes ratio and lower alpha diversity indices of microbiota [49]. Herein, the Firmicutes-to-Bacteroidetes ratio was favored by the *T. molitor* meal diet in *D. labrax* and *H. illucens*, and *M. domestica* meal diets in *S. aurata*, compared to the control diet, indicating the dietary-induced differential between and within species alteration of gut microbiota. In contrast to our results, Antonopoulou et al. [17] observed a reduction in the aforementioned ratio in *D. labrax*, following fish meal replacement with *T. molitor*. However, despite the higher percentage of *T. molitor* inclusion, the Firmicutes-to-Bacteroides ratio displayed a similar decline in *S. aurata*. Moreover, weight gain and the Proteobacteria-to-Bacteroidetes ratio, an indicator of fish health [72], were negatively correlated in *D. labrax* of the control diet. A significant reduction in the Proteobacteria-to-Firmicutes ratio in *O. mykiss* fed black soldier fly meal has been attributed to chitin [25]. The latter is a long-chain homopolymer of N-acetylglucosamine [73] that forms an essential structural component of the outer exoskeleton of insects and crustaceans [74], and has been previously described as a substrate for the growth of chitinolytic bacteria, including lactic acid bacteria [24]. Few studies have indicated that direct inclusion of chitin as a supplement in aquafeeds [75,76] or indirect inclusion through insect meal diets [24,25,77] modulates the composition of fish gut microbiota and favors the colonization of non-common bacteria able to metabolize chitin as a nutrient.

### 4.2. Enrichment of Beneficial Bacteria

Among the Firmicutes that displayed significant shifts in abundance were genera of lactic acid bacteria, a group commonly used as probiotics in fish feeds [78] for the preservation of a balanced microbial regime. Lactic acid bacteria are considered beneficial to the host by promoting nutrient digestion through microbial fermentation [79] and by suppressing pathogens’ adhesion and growth [80]. Herein, *S. aurata* exhibited enrichment in *Carnobacterium* and *Leuconostoc* following the *M. domestica* meal dietary treatment. It is interesting to note that carnobacteria are among the aforementioned chitinolytic bacteria, capable of decomposing chitin [76]. However, in contrast with our results, dietary inclusion of *H. illucens* and *T. molitor* in the diet of Siberian sturgeon (*Acipenser baerii*) lead to an increase in the total number of *Carnobacterium* [81], whereas dietary chitin supplementation in *Salmo salar* favors the growth of both *Carnobacterium* and *Leuconostoc* members [75]. In addition, *O. mykiss* fed *H. illucens* larva meal exhibit a significant reduction in Carnobacteriaceae percentage [26], which is contrary to our findings. Such differential inter- and intra-specific microbial shifts may be derived from differences related to life stage, rearing substrate, and processing of insects, in addition to microbiota adaptation in accordance with host metabolic requirements [16,24,68].

Dominant bacterial species tend to control the gut microbiota equilibrium, and thus guide adaptation to new environmental or dietary conditions through regulation of pivotal functions [82]. *Aeromonas* and *Pseudomonas* members, which are often reported in many carnivorous teleosts [83,84], were among the most abundant shared bacterial species in the lumen of *S. aurata* and *D. labrax*, respectively, whereas simultaneously an induced abundance was observed in response to dietary treatments with insect meals. Bacteria belonging to the aforementioned genera have been reported to contribute to teleosts’ digestion through secretion of several digestive enzymes [15,84,85]. Józefiak et al. [81] similarly report a count increase in *Aeromonas* spp. in juvenile *A. baerii* following a full-fat *H. illucens* meal. However, a *T. molitor* meal diet did not significantly alter the aeromonad population. Similarly, enrichment in bacteria belonging to the genus *Pseudomonas* has been reported in rainbow trout following a partially defatted *H. illucens* meal [24]. In contrast, *Pseudomonas* has been reported to undergo a significant population reduction in juvenile rainbow trout in response to dietary inclusion of *H. illucens* [25]. 

The concept of incorporating insects into fish feeds presupposes the ability to utilize nutrient ingredients. A key point that should be considered is the metabolic adaptation of teleosts to dietary changes [86,87], which is highly linked to gut microbiota [29,57,88] because the evolutionarily steady diet of fish led to a limited ability for adaptive regulation in the digestive system [89]. Herein, insect meal diets with a significant reduction in fatty acid absorption, i.e., *T. molitor* for *D. labrax* and *H. illucens* for *S. aurata*, were simultaneously those among the ones that led to a decrease in Firmicutes’ abundance. The inference of these findings may be broadened following the suggestions of Semova et al. [29], who noted that enrichment of Firmicutes is positively correlated with an increase in the competence of host enterocytes to absorb fatty acids.

### 4.3. Alterations in Potential Functionality of Microbial Communitites

According to metagenome prediction regarding the functionality of bacterial communities, glutathione metabolism showcased similar differentiation in all insect meal diets compared to fish meal in *D. labrax*. This particular thiol, which has a prominent implication in homeostasis maintenance and cell protection during oxidative and osmotic stresses, is produced by, among others, Cyanobacteria and Proteobacteria in terms of prokaryotes [90,91]. Thus, the decrease in glutathione metabolism may be due to the differentiated bacterial community that occurred in insect-fed *D. labrax*, whereas reduction in Proteobacteria abundance has been noted in all insect diets. Moreover, functional pathways related to carbohydrate metabolism, such as pentose and glucuronate interconversions, seem to be induced by the dietary inclusion of insect meals in *D. labrax*. As discussed above, this may be due to the contribution of gut microbiota in chitin digestion. In addition, it is worth mentioning that the reduction of the alpha-linolenic acid metabolism (which is observed in *M. domestica*-fed *D. labrax*) may be attributed to the decreased amounts of omega-3 fatty acids in the specific diet. Previous studies have observed a lower omega-3 fatty acid content in *M. domestica* larvae [35]. Furthermore, up-regulation of genes associated with arginine and proline metabolism in *D. labrax* fed the *T. molitor* diet in the present study may be attributed to the increased dietary percentage of proline [35]. In addition, the simultaneous enhancement of Enterobacteriaceae is in agreement with that of Faure et al. [92] in rats’ microbiota, following amino acid supplementation, which included proline.

Regarding *S. aurata*, changes in the potential bacterial community functions included a decrease in gene pathways responsible for vitamin metabolism in the *H. illucens* dietary group. This, in addition to the simultaneous reduction in many pathways of amino acid metabolism, may underline the nutrient insufficiency of the specific substitution. Furthermore, enrichment of infectious disease-related pathways (e.g., amoebiasis) reinforces the possibility that the specific insect meal diet may not be suitable for *S. aurata*. In addition, both *H. illucens*- and *M. domestica*-containing diets led to an elevation in the microbial degradation of xenobiotics. Although the increase in these pathways may be due to the microbial communities’ adaptation in order to degrade contaminants likely to be present in the seawater, xenobiotic-metabolizing enzymes have been reported to degrade compounds with similar chemical structures [93]. However, results from metagenome prediction tools should be interpreted with hesitation. Sun et al. [94] questioned the reliability of metagenome prediction tools and indicated the inconsistency of the inferences in non-human datasets due to the fact that genome databases are biased towards human-related microorganisms.

### 4.4. Diet-specific Microbial Interactions Driven by Insect Meal Inclusion

Assembled microbial communities are formed and evolved through microbe-to-microbe interactions (complementation, cooperation, competition, predation) which can drive evolutionary radiations and determine several functions of the microbiota [95]. Diet is a key factor in the configuration of stable and dynamic microbial communities [96]. As revealed by this study, the more complex interacting networks between genera in fish meal, *H. illucens* and *T. molitor* diets contrasted with the simpler network of the *M. domestica* diet. The presence of many interactions is known to be indicative of functionally dependent cooperative communities [97]. Although negative interactions may imply competition for substrates utilization [98], thus leading to a potential disruption of pivotal to the host microbial cooperative metabolism [99], microbial competition may benefit the host through stability promotion [100]. Diet-specific interactions were apparent in the network analysis performed in this study, indicating that different ingredients may require different biochemical processes that favor certain bacteria and differentiate the co-occurrence patterns. Fish meal substitution with insect meals favored the emergence of unique microbe-to-microbe correlations. The genera *Acinetobacter*, *Anaerococcus*, *Delftia*, and *Pseudomonas* contributed to many significant interactions that were present in the three networks of the insect-containing diets examined. However, to distinguish whether these patterns represent metabolite-driven interactions or coexistence in ideal regimes requires further research, in addition to network analysis [96,101]. Nonetheless, networks reflected the dietary-induced presence/absence of microbial coexistence, which, in addition to keystone species, establishes the dynamics of a microbial community [102].

## 5. Conclusions

The present study stressed the species-specific differential responses of the gut microbiota to nutritional changes and demonstrated that dietary inclusion of insects as partial fish meal substitution resulted in a partially different configuration of the gut structure of resident bacteria. Insect meals maintained the dominance of the major microbial phyla, while simultaneously may have opened new niches that probably favored the prevalence of species that are considered to be beneficial to teleosts’ health. Dominant bacterial species that may mediate teleost digestion were enriched following inclusion of insect meal in aquafeeds. In particular, inclusion of *M. domestica* enhanced beneficial bacteria, especially in *S. aurata*. Future fish feed design must take into account the nutritional requirements and trophic level of the teleost species. In addition to optimizing growth parameters, food composition should be conducive to maintaining a balanced microbial profile that is capable of contributing to host metabolism in a multifaceted manner. The results of this study pave the way in this direction.

## Data Availability

Raw sequencing data have been deposited in Short Reads Archive of the National Center for Biotechnology Information (NCBI) (http://www.ncbi.nlm.nih.gov/sra) under the accession numbers PRJNA521099 for *D. labrax* and PRJNA608666 for *S. aurata*.

## Supplementary Materials

The following are available online at https://www.mdpi.com/article/10.3390/microorganisms9040699/s1, Table S1: Ingredient composition (g kg^−1^) and estimated proximate analysis of experimental diets of *Dicentrarchus labrax* and *Sparus aurata*. Table S2: Growth performance parameters and somatometric indices of *Dicentrarchus labrax* and *Sparus aurata* fed control (FM) and insect meal-containing diets (HI, TM, MD). Abbreviations according to Table S1. Figure S1: Rarefaction curve analysis applied to bacterial operational taxonomic units (OTU), clustered at the 97% phylotype similarity level derived from gut samples (*n* = 12) of *Dicentrarchus labrax* and *Sparus aurata* fed control (FM) and insect meal-containing diets (HI, TM, MD). Abbreviations according to Table S1. Table S3: Normalized 16S rRNA gene sequencing results and diversity indices in the gut of *Dicentrarchus labrax* and *Sparus aurata* fed control (FM) and insect diets (HI, TM, MD). Abbreviations according to Table S1. Table S4: Spearman’s rho non-parametric correlations of richness and bacterial phylum ratios with growth performance parameters and somatometric indices in *Dicentrarchus labrax* fed control (FM) and insect meal-containing diets (HI, TM, MD). Abbreviations according to Table S1. Table S5: Spearman’s rho non-parametric correlations of richness and bacterial phylum ratios with growth performance parameters and somatometric indices in *Sparus aurata* fed control (FM) and insect meal-containing diets (HI, TM, MD). Abbreviations according to Table S1.
