# Supplementary material for: Configuration of Gut Microbiota Structure and Potential Functionality in Two Teleosts under the Influence of Dietary Insect Meals
Source: Microorganisms. 2021 Mar 28;9(4):699. doi: 10.3390/microorganisms9040699 (PMC8067204; doi:10.3390/microorganisms9040699)
Supplement: Supplementary file 1 [file microorganisms-09-00699-s001.pdf]

Materials and Methods

Experimental Conditions and Feed Formulation

The growth performance parameters and somatometric indices were calculated using the following equations:

Survival (%) = 100 x final number of fish / initial number of fish

Weight gain (WG, %) = 100 x [FBW (Final Body Weight, g) – IBW (Initial Body Weight, g)] / IBW

Specific growth rate (SGR, % day^{-1}) = 100 x [(ln FBW - ln IBW) / number of days]

Daily feed intake (DFI, % body weight day^{-1}) = [total dry feed intake (g) x 100] / [(IBW + FBW) x 0.5] x number of days

Feed conversion ratio (FCR) = total dry feed / (FBW-IBW)

Condition factor (CF) = 100 x [body weight (g) / total length^3 (cm)]

Hepatosomatic index (HSI) = 100 x (liver weight / body weight)

Mesenteric fat index (MSI) = 100 x (perivisceral fat weight / body weight)

Viscerosomatic index (VSI) = 100 x (viscera weight / body weight)

Relative gut length (RGL) = gut length (cm) / fish total length (cm)

Table S1. Ingredient composition (g kg^{-1}) and estimated proximate analysis of experimental diets of *Dicentrarchus labrax* and *Sparus aurata*.

	Dicentrarchus labrax				***Sparus aurata***			
	**FM**	**HI**	**TM**	**MD**	**FM**	**HI**	**TM**	**MD**
Ingredients								
Fishmeal	650	455	455	455	650	455	455	455
Insect meal	0	195	195	195	0	195	195	195
Fish oil	100	97	60	62	100	97	51	62
Wheat	175.5	163.5	183.5	184.5	161	149	188	169
Wheat Gluten meal	67	66	82	91	64	64	71	85
Premix	2.5	2.5	2.5	2.5	25	25	25	25
DL-methionine	5	9	10	3	0	4	4	2
Lysine	0	12	12	7	0	11	11	7
Composition								
Crude protein (%)	55.0	55.0	55.0	55.0	55.0	55.0	55.0	55.0
Crude fat (%)	16.2	15.2	15.2	15.2	16.2	15.2	16.2	15.2
Ash (%)	12.7	10.5	10	10.5	13.3	11.1	10.4	11.1
Crude fiber and Carbohydrates (%)	16.1	19.3	19.8	19.3	15.5	18.7	18.4	18.7
GE (Mj/kg)	22.1	22.3	22.4	22.3	22.0	22.2	22.1	22.2
Lysine (%)	5.1	5	5	5	5.2	5.2	5.2	5.2
Methionine (%)	2.6	2.6	2.6	2.6	2.8	2.8	2.8	2.8

FM: Fish meal; HI: *Hermetia illucens*; TM: *Tenebrio molitor*; MD: *Musca domestica*.

Table S2. Growth performance parameters and somatometric indices of *Dicentrarchus labrax* and *Sparus aurata* fed control (FM) and insect meal containing diets (HI, TM, MD). Abbreviations according to Table S1.

	Dicentrarchus labrax				***Sparus aurata***			
	**FM**	**HI**	**TM**	**MD**	**FM**	**HI**	**TM**	**MD**
**Growth performance parameters**								
**IBW (gr)**	5.73±0.03	5.71±0.06	5.70±0.04	5.70±0.07	29.74±0.09	29.37±0.23	29.08±0.24	29.80±0.09
**FBW (gr)**	22.40±1.52	22.21±1.65	19.93±1.29	22.78±1.07	121.66±5.05	123.67±4.31	131.46±3.66	124.56±3.50
**WG (%)**	290.14±25.40	289.62±30.01	248.62±21.19	301.76±22.23	308.84±16.46	321.76±16.01	352.49±13.57	317.99±11.80
**FCR**	1.20±0.10	1.38±0.17	1.40±0.22	1.09±0.07	1.08±0.07	1.10±0.06	1.00±0.04	1.07±0.04
**Somatometric indices**								
**CF**	1.08±0.03	1.10±0.02	1.05±0.02	1.06±0.01	1.56±0.03^{b}	1.61±0.03^{ab}	1.71±0.02^{a}	1.69±0.11^{ab}
**HSI (%)**	1.65±0.17	1.76±0.10	1.76±0.10	1.76±0.13	1.26±0.05	1.41±0.11	1.49±0.07	1.37±0.05
**VSI (%)**	8.65±0.68	10.55±0.43	9.95±0.46	10.06±0.44	5.46±0.32	5.90±0.31	5.93±0.26	5.81±0.22
**MFI (%)**	3.71±0.34	4.83±0.32	4.23±0.32	4.35±0.32	0.86±0.13	0.90±0.12	0.85±0.06	0.87±0.11
**RGL**	0.82±0.04	0.73±0.03	0.77±0.04	0.77±0.04	1.65±0.05	1.84±0.08	1.86±0.04	1.78±0.07

IBW: initial body weight; FBW: final body weight; WG: weight gain; SGR: specific growth rate; DFI: daily feed intake; FCR: feed conversion ratio; CF: condition factor; HIS: hepatosomatic index; VSI: viscerosomatic index; MFI: mesenteric fat index; RGL: relative gut length.

^{a,b}Different superscript letters in the same row indicate statistically significant differences as determined by one-way ANOVA, followed by Tukey's post hoc test. Statistical significance assessed at the 0.05 level.

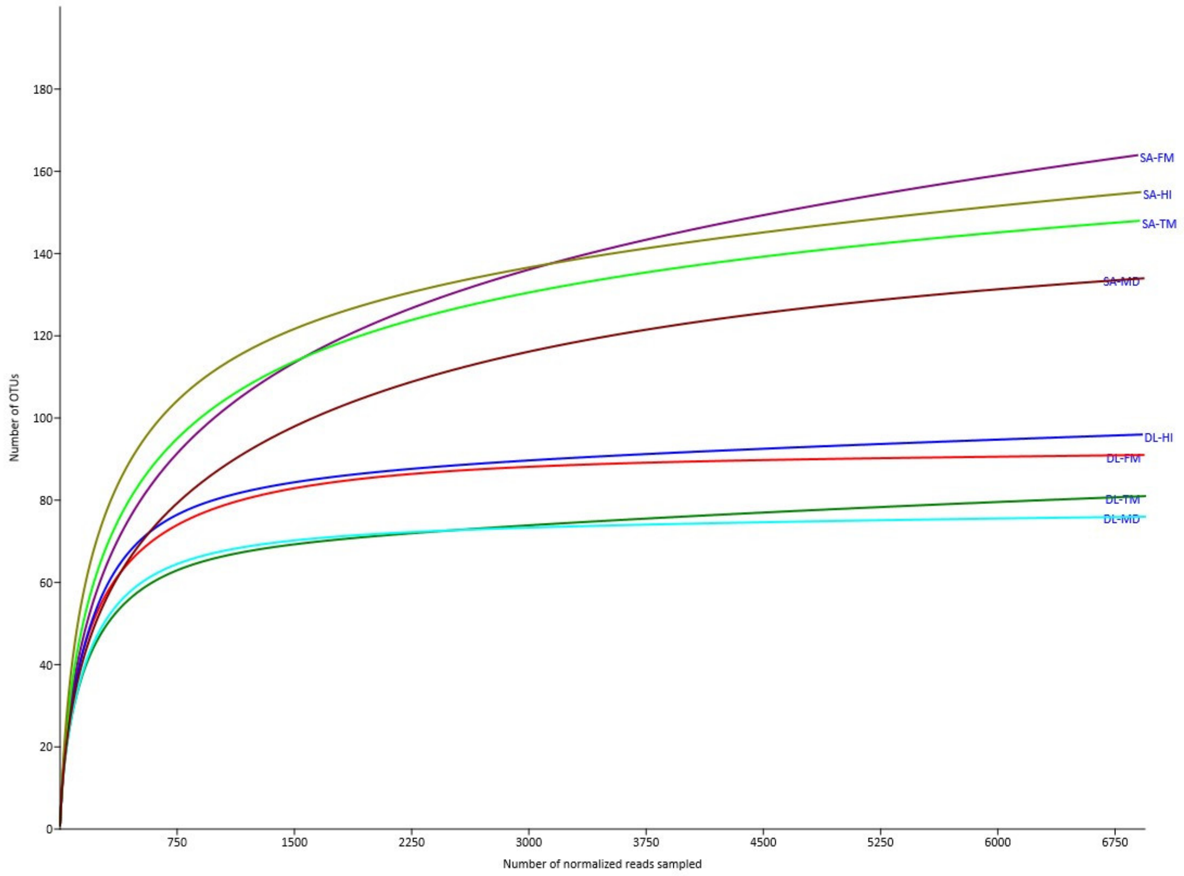

Figure S1. Rarefaction curve analysis applied to bacterial operational taxonomic units (OTU), clustered at the 97% phylotype similarity level, that derived from gut samples (n=12) of *Dicentrarchus labrax* and *Sparus aurata* fed control (FM) and insect meal containing diets (HI, TM, MD). Abbreviations according to Table S1.

Table S3. Normalized 16S rRNA gene sequencing results and diversity indices in the gut of *Dicentrarchus labrax* and *Sparus aurata* fed control (FM) and insect diets (HI, TM, MD). Abbreviations according to Table S1.

	**Diet**	**Reads**	**OTUs**	**Dominance of the most abundant OTU**	**No. of most dominant OTUs***	**Shannon diversity index**	**Chao1 richness estimator**
Dicentrarchus labrax	FM	7001±0.9	148±7.2^{a}	13.3	24 (80.0%)	2.60±0.070	137±14.2
	HI	7001±1.0	142±6.3^{ab}	11.8	25 (80.2%)	2.60±0.071	123±11.3
	TM	7003±1.5	129±4.5^{b}	14.2	19 (80.6%)	2.49±0.078	120±10.8
	MD	7004±1.1	124±6.8^{b}	14.3	19 (80.0%)	2.32±0.084	132±13.6
Sparus aurata	FM	7005±2.5	99±9.9^{ab}	12.8	27 (80.4%)	2.40±0.199	89±11.0^{a}
	HI	7013±2.4	74±9.3^{ac}	8.1	40 (80.1%)	2.65±0.099	107±38.3^{a}
	TM	7005±0.9	103±7.4^{b}	9.2	32 (80.3%)	2.51±0.141	72±6.5^{a}
	MD	7010±1.5	58±2.3^{c}	17.5	21 (80.6%)	2.50±0.090	43±4.9^{b}

*: cumulative relative abundance of ≥80%.

^{a,b,c}Different superscript letters indicate statistically significant differences between dietary treatments in *D. labrax* or *S. aurata* as determined by Mann-Whitney test for multiple comparison. Statistical significance assessed at the 0.05 level.

Table S4. Spearman's rho non-parametric correlations of richness and bacterial phylum ratios with growth performance parameters and somatometric indices in *Dicentrarchus labrax* fed control (FM) and insect meal containing diets (HI, TM, MD). Abbreviations according to Table S1.

	**Proteobacteria:Actinobacteria**				**Proteobacteria:Bacteroides**			
	**FM**	**HI**	**TM**	**MD**	**FM**	**HI**	**TM**	**MD**
**WG**	-0.22	0.23	0.03	-0.14	**-.664***	-0.15	0.15	0.34
**FCR**	0.22	-0.23	-0.08	0.21	**.669***	0.10	-0.16	-0.32
**VSI**	0.18	0.09	**.684***	0.00	-0.40	-0.31	-0.29	-0.22
**MFI**	-0.11	-0.27	**.815****	-0.13	-0.20	0.17	0.13	-0.18
**RGL**	0.13	-0.01	-0.36	-0.02	0.46	0.14	0.26	0.15
	**Proteobacteria:Firmicutes**				**Firmicutes:Actinobacteria**			
	**FM**	**HI**	**TM**	**MD**	**FM**	**HI**	**TM**	**MD**
**WG**	-0.13	**.608***	0.12	0.48	-0.03	-0.27	0.03	-0.55
**FCR**	0.13	**-.676***	-0.16	-0.47	0.06	0.28	-0.07	**.580***
**VSI**	0.17	-0.13	0.25	**-.664***	0.19	0.20	0.33	0.50
**MFI**	-0.30	-0.25	0.51	-0.54	0.41	-0.10	0.28	0.37
**RGL**	-0.01	**.720****	-0.12	-0.19	0.30	**-.678***	0.01	0.19
	**Firmicutes:Bacteroides**				**Richness**			
	**FM**	**HI**	**TM**	**MD**	**FM**	**HI**	**TM**	**MD**
**WG**	**-.650***	-0.45	0.09	0.02	0.01	**.624***	0.15	**.587***
**FCR**	**.648***	0.40	-0.09	-0.01	-0.03	**-.712****	-0.16	-0.57
**VSI**	-0.39	-0.24	-0.31	0.08	0.13	0.05	0.34	-0.21
**MFI**	-0.10	0.27	-0.19	0.03	-0.12	0.22	**.620***	-0.15
**RGL**	0.20	-0.17	0.54	0.20	0.16	**.631***	-0.20	-0.06

GL: gut length; WG: weight gain; SGR: specific growth rate; DFI: daily feed intake; FCR: feed conversion ratio; VSI: viscerosomatic index; MFI: mesenteric fat index; RGL: relative gut length.

**. Correlation is significant at the 0.01 level (2-tailed).
*. Correlation is significant at the 0.05 level (2-tailed).

Table S5. Spearman's rho non-parametric correlations of richness and bacterial phylum ratios with growth performance parameters and somatometric indices in *Sparus aurata* fed control (FM) and insect meal containing diets (HI, TM, MD). Abbreviations according to Table S1.

	**Proteobacteria:Actinobacteria**				**Proteobacteria:Bacteroides**			
	**FM**	**HI**	**TM**	**MD**	**FM**	**HI**	**TM**	**MD**
**WG**	-0.09	0.24	-0.10	-0.02	-0.45	-0.40	0.40	-0.02
**FCR**	0.14	-0.23	0.07	-0.01	0.48	0.39	-0.43	0.10
**VSI**	-0.08	0.35	-0.03	0.45	-0.21	-0.42	0.28	-0.12

**MFI**	0.34	0.43	-0.56	0.34	0.12	-0.33	-0.10	0.12
**RGL**	0.28	0.35	-0.02	0.00	**-.685***	0.00	-0.37	0.24
	**Proteobacteria:Firmicutes**				**Firmicutes:Actinobacteria**			
	**FM**	**HI**	**TM**	**MD**	**FM**	**HI**	**TM**	**MD**
**WG**	-0.04	0.26	-0.57	-0.08	0.03	0.01	0.36	-0.11
**FCR**	0.09	-0.27	**.596***	-0.02	-0.02	0.00	-0.35	0.12
**VSI**	**-.650***	0.25	-0.43	0.22	0.31	0.00	0.29	0.31
**MFI**	**-.601***	0.47	**-.655***	0.50	**.657***	-0.06	-0.32	-0.01
**RGL**	0.10	-0.21	0.21	-0.36	0.18	0.43	-0.01	0.01
	**Firmicutes:Bacteroides**				**Richness**			
	**FM**	**HI**	**TM**	**MD**	**FM**	**HI**	**TM**	**MD**
**WG**	-0.55	-0.45	0.55	0.17	0.34	0.12	**-.757****	0.44
**FCR**	0.56	0.43	-0.59	-0.08	-0.32	-0.10	**.712****	-0.43
**VSI**	0.03	**-.683***	0.45	0.00	0.09	0.41	-0.24	0.10
**MFI**	0.30	-0.54	0.07	0.12	0.06	0.09	0.00	0.11
**RGL**	-0.52	-0.28	-0.42	0.29	**.615***	0.20	-0.14	0.15

GL: gut length; WG: weight gain; SGR: specific growth rate; DFI: daily feed intake; FCR: feed conversion ratio; VSI: viscerosomatic index; MFI: mesenteric fat index; RGL: relative gut length.

**. Correlation is significant at the 0.01 level (2-tailed).
*. Correlation is significant at the 0.05 level (2-tailed)
